# Choosing to live with home dialysis-patients' experiences and potential for telemedicine support: a qualitative study

**DOI:** 10.1186/1471-2369-13-13

**Published:** 2012-03-19

**Authors:** Ellen Rygh, Eli Arild, Elin Johnsen, Markus Rumpsfeld

**Affiliations:** 1 Norwegian Centre for Integrated Care and Telemedicine, University Hospital of North Norway, PO Box 35, N-9038 Tromsø, Norway; 2 Division of Internal Medicine, University Hospital of North Norway, PO Box 100, N-9038 Tromsø, Norway

**Keywords:** Home dialysis, Modality selection, Fellow patients, Telemedicine, Chronic care model, Patient empowerment, Self-management

## Abstract

**Background:**

This study examines the patients' need for information and guidance in the selection of dialysis modality, and in establishing and practicing home dialysis. The study focuses on patients' experiences living with home dialysis, how they master the treatment, and their views on how to optimize communication with health services and the potential of telemedicine.

**Methods:**

We used an inductive research strategy and conducted semi-structured interviews with eleven patients established in home dialysis. Our focus was the patients' experiences with home dialysis, and our theoretical reference was patients' empowerment through telemedicine solutions. Three informants had home haemodialysis (HHD); eight had peritoneal dialysis (PD), of which three had automated peritoneal dialysis (APD); and five had continuous ambulatory peritoneal dialysis (CAPD). The material comprises all PD-patients in the catchment area capable of being interviewed, and all known HHD-users in Norway at that time.

**Results:**

All of the interviewees were satisfied with their choice of home dialysis, and many experienced a normalization of daily life, less dominated by disease. They exhibited considerable self-management skills and did not perceive themselves as ill, but still required very close contact with the hospital staff for communication and follow-up. When choosing a dialysis modality, other patients' experiences were often more influential than advice from specialists. Information concerning the possibility of having HHD, including knowledge of how to access it, was not easily available. Especially those with dialysis machines, both APD and HHD, saw a potential for telemedicine solutions.

**Conclusions:**

As home dialysis may contribute to a normalization of life less dominated by disease, the treatment should be organized so that the potential for home dialysis can be fully exploited. Pre-dialysis information should be unbiased and include access to other patients' experiences. Telemedicine may potentially facilitate a communication-based follow-up and improve safety within the home setting, making it easier to choose and live with home dialysis.

## Background

Since patients play a vital self-management role in home dialysis, it is essential to understand what is important to them when deciding on a dialysis modality. Several studies have shown that if patients are actively involved in the process of choosing their treatment modality, there is a greater likelihood that they will choose PD [1]. This requires unbiased pre-dialysis counseling, close consideration of patients' preferences and life situation, as well as easy access to health professionals and technical support [2].

The treatment of end-stage renal disease (ESRD) varies nationally and internationally. Norway has one of the world's highest transplant rates (60.5 per million inhabitants) [3]. There is a 5% yearly growth in the prevalent dialysis population [3,4]. Hospital in-center haemodialysis (CHD) is the most frequent dialysis modality (81%), while the PD rate is relatively low (19%) [4]. Other Scandinavian countries have higher rates for home dialysis, for instance Denmark (30%) [2,3]. This finding indicates a potential for increasing the rate of home dialysis in Norway. For patients who cannot undergo a transplant, and for those awaiting a transplant, PD is considered the best and cheapest treatment [5]. Survival in patients on PD is at least as good as, and sometimes even better, than CHD [6]. Based on the growing numbers of patients suffering ESRD and increased pressure on the rental units, there is an initiative in Norway for increasing the rate of home dialysis [7].

For many years PD has been the preferred home treatment in Norway, but it is not suitable for everyone. It is therefore appropriate to concentrate more on HHD [8]. In New Zealand and Australia, 15.5% and 9.8% of dialysis patients use HHD, whereas the rate in Norway is 0.3% [3,9]. HHD creates the opportunity for more frequent treatment and increased and individualized dialyzing time, which leads to improved blood pressure control, less diet restrictions and less need for medication. This provides for better preservation of residual renal function and improved survival [10,11].

There are few reports on the use of telemedicine in dialysis treatment in general, and in home dialysis specifically. Telemedicine may be defined as "...*the use of electronic information and communications technologies (ICT) to provide and support health care when distance separates the participants" *[12]. In Norway, decentralized hemodialysis is some places given in "satellite centers" without a nephrologist, and telemedicine solutions have been successfully designed for this purpose [13]. There are some reports of follow-up of home dialysis patients by telephone or video conference (VC), which allow for closer support and problem-solving from a distance [14-17]. Remote monitoring of nightly HHD is practiced in the United States, Canada and the Netherlands, and consists of transfer of dialysis data and alarms over phone or the Internet to a service center [18,19].

Our study aims to understand the patients' need for information and guidance in the course of modality selection, training and establishing of home dialysis. The study is based on a material concerning how patients experience living with home dialysis, how they master this complex and advanced treatment, how they live their everyday lives, and their views on the optimization of information and communication with health care.

## Methods

The empirical basis of this study is in-depth interviews with eleven patients established in home dialysis, eight PD- and three HHD-patients. The interviews were conducted in autumn 2008. All patients on PD affiliated with the University Hospital of North Norway (UNN) were eligible, and inclusion criteria were whether they were well established in home dialysis and fit physically and/or mentally to be interviewed and visited in their home. The patients were recruited by the dialysis nurses. There were sixteen patients on PD at the time; nine were included for interview. Six patients were considered not capable to participate: one patient was demented, one had psychological problems, and four had serious complications or were in a palliative phase. One patient refused to participate. One included patient got acutely ill and was hospitalized when we arrived at his home. In addition the material comprises all known HHD-users in Norway at that time, one within and two outside UNN's catchment area. The interviewers did not have any relationship with the patients beforehand. Seven of the interviews took place in the informants' homes; three were for practical reasons interviewed in the hospital and one by telephone. The home visits gave the opportunity to observe how the patients organized the dialysis in their home environment and how they lived their daily life.

An interview guide with open questions was used (Table 1: Interview guide).

**Table 1:** Interview guide

i.	Questions about demographic data
	[Age, partner or spouse, other close relatives, dwelling, work, distances to hospital and health care center]

ii.	Describe your medical history

	[When did your kidney disease start; how old were you then; how long have you had the dialysis at home; have you had other dialysis modalities; have you had kidney transplants; in case how many]

iii.	Describe how your communication with the health care services functions

	[Where do you go for follow-up and how often; do you have contact with other specialists, your GP or other parts of primary health care; in case how are these informed about your kidney disease and the dialysis treatment, and do you think that they can help you if needed]

iv.	Describe your experiences living with home dialysis

	[How is your general experience with home dialysis; do you feel safe or is there something that makes you anxious or uncomfortable; have you had any technical problems with the equipment, whom do you contact then to get help; have you had any medical complications or emergencies, whom have you then contacted for help]

v.	Describe how it was to choose between the different treatment options

	[How did you get information, from doctors, nurses, patient association, other patients, other info; what were the decisive factors for choosing this form of dialysis; did you find the information you received adequate; was there anything that could make you safer if you were unsure; do you feel that you made the right choice, or would you have chosen differently now that you know more; what could in case helped you to make other choices]

vi.	Describe how was the training in home dialysis

	[Where did it take place, what kind of staff was responsible; what was good and what was less good with the training; how long were you in hospital for the training; how did you feel when you were ready to go home, were you confident or anxious, in case for what reasons; what could you want differently or in addition to the information and training that you got]

vii.	Describe what it was like when you got home and had to do the dialysis on your own

	[Did you need help from others in your daily life to cope with the dialysis, for instance family members, home visiting nurse service; was there any follow-up that you needed]

viii.	General knowledge and use of technologies in daily life

	[Questions and observations about competence in use of ordinary technological equipment like mobile phones/personal computers; attitude towards using new/unfamiliar technology]

ix.	What could potentially make the home situation with dialysis easier and safer with telemedicine?

	[Would this make it easier to choose home treatment over hospital dialysis; what is the preconditions to take such technology into use, (training, helpdesk); in what situations would you see a possible use; who would you prefer to contact; for how long do you think it appropriate to have the equipment and service available]

The interviews were conducted by two persons; one person performed the interview and the other composed field notes. The interviews were audio-taped and transcribed verbatim, except for the HHD interviews, which lasted for several hours. One interview with a PD informant is also based on the notes alone because the tape recording failed. In these cases the interviewers listened through the recordings once again during the analysis process.

### The interviewees

The interviewees were eight patients with PD; three with APD and five with CAPD. Three patients had HHD; these represented all known patients with HHD in Norway at that time. Only two of our informants had CAPD as their primary and only modality. The other informants had experience with various forms of dialysis, and many had changed modality several times.

The interviewees with HHD were two women and one man, aged 36-60 years, all working. They spent four to five days, 16 to 20 hours a week, on dialysis. All had received three previous transplants. One had previously practiced APD for two years; this was stopped for medical reasons. Two informants had performed HHD for more than two decades, the last for about three years. One managed the dialysis without any assistance; the two others relied on friends or family for practical support and security. One, who had a definite need for back-up, had designed (together with the hospital staff) a telemedicine connection to the local hospital's emergency department, which was able to react to machine alarms.

The interviewees with PD were two women and six men, aged 23-82 years; five were over 60 years old. Two were working and the rest were retired or had disability pensions. All lived in their own home. One informant with APD had assistance from a visiting nurse service; the others performed the dialysis procedures alone. Only two had previously received transplants; three were waiting for transplants.

### Ethical considerations

The project was submitted for the Regional Ethics Committee who found that their approval was not necessary. The study was approved by the Chief security officer at the University Hospital of North Norway, who ensures that personal information is treated lawfully and safely. All participants gave their voluntary, informed and written consent to participate in the project, including consent to publication of the results in a scientific journal.

### Analysis

Analysis is mainly based on an inductive research strategy, searching the actor's point of view through interview data and observations [20]. Our focus was the informants' experiences, and our theoretical frame of reference was patient empowerment and self-management through ICT solutions [21].

Analysis of the material was performed in the following stages: (i) the interviewers read (or listened to) all the collected material to obtain an overall impression; (ii) units of meaning were identified and coded in a matrix according to the questions in the interview guide; (iii) this material was further structured and condensed into more general patterns, and (iv) findings were summarized into major themes [22].

## Results

Visiting informants in their home, we could observe how dialysis influenced their everyday activities. One informant had just come home from moose hunting. Another had installed himself for the CAPD procedures by the kitchen table, which offered the best view in the house; and one had arranged a home hospital in the basement living room, where helpers and friends regularly paid visits.

Major themes that emerged from the interviews were: (i) choice of modality; (ii) training and coping; (iii) communication with health services; (iv) quality-of-life and sick role; (v) potential for telemedicine.

### Choice of modality

All of our interviewees expressed satisfaction with the choice of home dialysis. Many pointed out that they could have wanted information about the possibility of home dialysis, as well as information about different modalities, at an earlier stage. Observation of and listening to other patients' experiences were often more influential on their choice than information from health professionals:

- ... *all those with haemodialysis, they are in fact mostly bedridden, being transported down three times a week, and completely exhausted when they come back, and they say they are in pain afterwards, too. ... I talked to several people who were in hospital just for controls of that PD-dialysis. One, an old man of 80 years, told that he took the equipment along when he was in the forest chopping wood ... (PD patient)*

- *They, the doctors, can explain, so and so is it, but they cannot tell how you experience it. Oh, it was really good to talk with others who have experienced the same thing. I think this could be a lot of the reason that you make a choice, really ... (PD patient)*

The interviewees had individual reactions to the different modalities, according to their personal preferences and life situation:

- ...*the nice thing with the [night] machine is that you get the day free, then you can do whatever you want. But there is a slight catch to it, to get these catheters to work ... (PD patient)*

- *I am young you know; we are out at night now [in summer], and I had to go home and arrange my stuff. I was just pissed off with the machine; honestly, it was puffing and blowing; I was lying there, and felt maybe I was caught by it. No, I just couldn't manage that... (PD patient)*

The HHD users had all discovered this modality by chance, and they had to make quite hard efforts and actively request the hospital to acquire the competence to deliver this alternative to them:

- ... *If I had continued with hospital dialysis, I had been dead ... I realized that I had to take control of the situation, otherwise I would not bear to live. In part, I would die from physical diseases, in part I would die of depression ... The doctors were willing, but the method was almost forgotten. They were a little shaky, the procedure had to be invented all over again ... (HHD patient)*

### Training and coping

Nearly all the respondents felt they had received sufficient training in the hospital. They found the treatment easy to learn and manage, and after a while they mostly felt safe in the home setting.

- ...*so after that week there, then I had it all in my head, it was no problem. Yes, then it all worked automatically. (PD patient)*

- ....*was initially a bit sceptical; thought it was worse than it really was. (PD patient)*

- *It was just to get home and get away from the hospital. It was just to take the bag and hang it on the wall ... (PD patient)*

- *The first ten times, the tubes and all are a total chaos, but once you have practiced for a while, it's not that hard ...(HHD patient)*

In contrast to this, one of the older informants with APD felt insecure and did not like being alone at night. This feeling was strengthened when, in the beginning, neither the informant nor the community nurses were able to manage the machine alarms:

- *They say the treatment is harmless, but you're taught to be afraid of electricity. I'm safer now, but jump when the alarm goes ... (PD patient)*

After a time at home the interviewees developed a considerable ability to manage their own illness, and were often so independent that they could react to complications and even suggest adjustments in the treatment:

- *My situation is incredibly more stable and calm than what is usual in the hospital ... three hours of dialysis is no problem, no stress... Eventually you get to understand how much fluid to remove, the correlations... [You] get responsibility and then understand more. With more frequent dialysis one does not need to remove as much fluid. The chance of blood pressure fall is almost gone; it's almost like a jog... (HHD patient)*

- ...*have been ill for many years and know the symptoms in my body clearly. The blood pressure may vary by the amount of fluid removal, but [I] have not had blood pressure fall at home. Must be stable to take HHD, [can get] cramps in my legs, [be] dizzy and ill. Must be familiar with the symptoms, but have not had any of this at home, I stop the ultrafiltration before it comes that far ... (HHD patient)*

### Communication with health services

The feelings of security and coping when coming home were closely related to satisfactory access to professionals in the dialysis unit. The informants required very close contact with the hospital staff for issues concerning machine alarms, complications and related diseases, but also routinely for practical things like ordering materials. This was a very personal relationship, where the patient had his own nephrologist over time and had a direct number to the hospital department. The dialysis nurses knew practically everyone by their voice; it was enough to call and say, "Hey, it's me."

-...*Important that the kidney doctor is involved in everything that happens. It is a clear precondition for optimal treatment of chronic dialysis patients to have an open door to the hospital. (HHD patient)*

-*I know the nurses, no matter who I call, they know me. They have been very supportive ... (PD patient)*

Most had little contact with primary health services and with their general practitioner (GP), so that it was the nephrologist who functioned as their GP also in cases of minor intercurrent diseases. This was partly because many had developed knowledge about their disease that exceeded the competence of the GP.

-*And he [the kidney doctor] has taken care of nearly everything, so I don't have to go to the GP... (PD patient)*

-*The GP and I agree that it is I who am the specialist... (HHD patient)*

### Quality of life and sick role

The alternative to home dialysis was CHD, which would occupy four hours, three days a week in addition to the travel time needed. Informants felt that having to be dependent on the hospitals' opening hours would be a significant obstacle to living a normal life with regard to work and leisure time.

-...*super important to have had home dialysis, have completed education, [have been] financially independent, had an almost full and normal life. Have worked, taken care of myself, controlled my own time. [This] has done that I have kept healthier; [I] had been on a completely different planet if I had received disability pension 18-years-old. (HHD patient)*

Those who had received CHD earlier, told that home treatment provided a considerably improved quality of life. Many felt in better shape and had less medical complications:

-... *from lying in the hospital three days a week with that hemodialysis and being ill and in bad shape and everything, and repetitions on the drinking and eating and stuff. When I changed to this type of dialysis, it was quite a different everyday life for me. (PD patient)*

-...*when I now see how much better I am, and how much better I function in everyday life, i find it somewhat strange, that there are not more of the patients that go for the system that I have. (PD-patient)*

-...*got a very different life when I changed from hemodialysis to PD. Especially after I got the night machine it was much easier, you can utilize the day better; not a problem to be fully employed... (PD patient)*

Despite having a very serious illness and the fact that they spent a lot of their time on dialysis, many perceived themselves only to a limited extent as ill. It seems that being able to take control of their lives also helped them not feel like patients:

-*When I take the treatment myself, I shelter myself from being a patient... (HHD patient)*

The time outside the dialysis was too valuable to be wasted on long journeys to and stay in the hospital. In addition, in the hospital, they experienced how sick many of the other patients were; which was an uncomfortable reminder that this might be their fate also:

-... *stressing to get to know so sick patients, see that young people are dying, disgusting to be reminded that so sick can I be. [I have] not seen myself as sick ... (HHD patient)*

### Potential for telemedicine

Informants felt that routine admissions to the hospital were a strain, and they suggested that follow-up could be conducted by telecommunication.

-... *don't have to travel to the city; to spend 10 minutes, instead of as now, to spend a whole day. (PD patient)*

The six persons on dialysis machines (APD and HHD) were most responsive to telemedicine guidance and follow-up in their home, believing that this could enable more patients to have dialysis at home:

-...*so it had been ingenious to have that camera function stuff, then one can actually see....because it is not as easy to explain everything over the phone. It gives a feeling of safety [when] somebody observes that everything is going as it should. (PD patient)*

Those with HHD were especially positive to the potential of ICT solutions, like transmission of data and remote monitoring and problem-solving over VC:

-... *a central unit with a webcam to monitor the on-and off-coupling procedures; for that is what people are afraid of. Then even the most nervous and insecure persons can handle this. (HHD patient)*

-... *with videoconference the responsibility of the dialysis user will not be so heavy as it is today. I would also think that it could be less scary to start up, when you are not alone. .... could [then] be easier to motivate for home hemodialysis ...(HHD patient)*

Those without machines did not see advantages of telemedicine. For instance, most of the respondents with CAPD, generally the older men, were satisfied to use their mobile phone when needing to call the hospital department:

-...*I think it's best to explain through the phone. (PD patient)*

-...*I write it down in a form, and eventually read it loud for them on the phone. [I] will not have it transmitted on data, I'm too old to do things like that... (PD patient)*

-...*had it been as easy as just turning on the TV, then it hadn't been a problem... (PD patient)*

## Discussion

### Validity

Our preconception was that telemedicine may be useful in supporting patients doing dialysis in their homes, thus making it easier for more patients to choose and live with home dialysis. This could, as a result, lead to enhanced quality of life and better treatment for more patients. Many of our interviewees had no previous experience with telemedicine. We therefore had to explain what this concept implied. They, in turn, responded to their experiences with home dialysis without telemedicine, and their imagination of what telemedicine could add. Several informants, especially those without machines, did not see any added value of telemedicine, which suggests that they did not feel obliged to please us. Others, mainly respondents using dialysis machines, saw benefits in telemedicine and ICT. This may indicate that they were more familiar with the use of technology.

### Sample size and selection

This study represents comprehensive in-depth interviews of a purposeful sample of PD-patients in the catchment area of the University Hospital of North Norway, and all known HHD users in Norway at the time the study was conducted. The small sample size reflects the limited population of 230 000 inhabitants in this area. During 2008 the number of patients receiving PD fluctuated from sixteen patients in January to eight patients in December (4). There is no reason to believe that our results and conclusions would be different if we had interviewed more of the excluded patients. Although data saturation was not a question, since we interviewed all the feasible patients, we found our material robust and diverse, appropriate to answer the research questions of this study. The observations of the patients in their homes gave additional insight into informants' everyday life. The fact that all PD users came from the same catchment area, may imply that the pre-dialysis information may not be the same as for other areas where this is organized differently. However, as a whole the responses were consistent in illuminating patients' needs regarding choice of modality and their experiences from living with home dialysis, which makes the study transferable to this patient group on a larger scale.

### Choice of dialysis modality

One main result of this study is the informants' satisfaction with their choice and their competence in the performance of home dialysis. It is also noteworthy that the possibility of having HHD was hard to access, confirming that the art of HHD has more or less been forgotten in Norway [3,8]. It is important that information is provided so that users can make an educated choice in coherence with both their preferences and their options [23,24]. We assume that most dialysis units try to practice this in line with accepted guidelines [25]. However, treatment traditions, availability, capacity and staff skills are factors likely to influence the availability of choices. Particularly users starting with CHD often do not receive a choice of other modalities, and once established in a modality, they are seldom inclined to make a change [1,2].

It was crucial for many of our informants to observe and speak with fellow patients who were using various forms of dialysis. However, these encounters occurred at random while they were in the hospital. The same is reported from Denmark, where patients wanted to meet peers before making a choice [2]. These fellow patients may be seen as "lay experts" and should be used more systematically when informing and educating new patients [26]. There is, however, a discussion whether patient-to-patient education may bias new patients' choices of modality [27]. In our view also "objective", professional information may be biased; the doctor's advice reflecting his particular professional traditions and attitudes. The HHD-patients' experience, that this modality had been so hard to access, is one example of this. It is a challenge to build in access to the peer competence in the hospitals' organization. This may be organized, for example, by patient schools, and for practical reasons also by help of VC or web meetings.

### Training and coping

The informants found the treatment surprisingly easy to learn and to master, and most felt safe in their home situation as long as they had easy access to the hospital staff by phone. Self-administered home dialysis requires that the users follow complex treatment regiments, monitor their condition and make decisions about treatment adjustments, deciding when to seek help and when to handle a problem on their own. Thus they become responsible for their daily management in a "collaborative partnership" with the hospital staff [28]. Many of the interviewees in our study actually had a role that may be labeled as "co-specialists". Indeed, they appeared to have a unique knowledge of their illness, their body and of the management of their own life.

### Communication with health services

The interviewees needed a very close link to the hospital to feel secure as self-managers. How this "umbilical cord" to the expertise should work in terms of organization, is a relevant issue for further discussions. With increasing use of home dialysis, more tasks may be transported to the primary health services, and in small municipalities where ESRD patients are rare, health care personnel will have little experience with these patients. Telemedicine may then represent a potential for remote support.

The health care system for chronic conditions in general has come into focus, and models for chronic care are described [21,28-31]. Several of the components described as key elements in the "Chronic Care Model" are of relevance to patients with ESRD: Individual follow-up by informed health care personnel; decision support; and a proactive care plan on how to meet complications. A central element is "the informed, activated patient," requiring a shift in focus from traditional didactic patient information to patient empowerment and self-management skills [28,32].

We believe, however, that it is important to distinguish between the fundamental differences in roles and responsibilities of the patient and the professional: "Patient empowerment" and "self-management" should not lead to excessive burdens and responsibilities on the patient [33,34]. In this context, telemedicine may contribute to strengthened self-management and increased patient empowerment, respecting the differences in roles: This happens when telemedicine improves the conditions of home-based dialysis by improving access to professional expertise and supporting a communication-based follow-up.

### Quality of life and sick role

Even though the dialysis program was time consuming and controlled much of their lives, the informants did not perceive themselves as being ill. They did not like being brought back into the patient role, such as during admissions in hospital for routine controls. It seems that mastering the treatment at home also brings along mastering the perceptions of the illness. This is also reported in Denmark: the patient role is not in keeping with the desire to live as normally and independently as possible, which is one of the main reasons for choosing home treatment [2]. Others have also found that illness perceptions are linked to quality of life in ESRD patients [35]. One striking feature in our study was the impressive life-stories of many of the informants when they compared hospital to home dialysis. On this basis we find it appropriate that decision makers design both the pre-dialysis information and the dialysis treatment so that the potential for home dialysis can be exploited optimally.

### Potential for telemedicine

As mentioned earlier, the potential of telemedicine solutions was a hypothetical question, as the informants had little experience with this tool. According to many of the informants, telemedicine solutions may potentially help create security for themselves and others who otherwise could not choose home dialysis. This was especially applicable to the informants using dialysis machines. However, we assume that in the future more patients doing home dialysis may benefit from telemedicine for communication, training, and security. There are available systems enabling the transmission of dialysis data, blood pressure and weight to the hospital staff [36]. VC may be useful for psychosocial support and for sorting out technical problems, and web based services may help gain access to informational material, and for patients to share their experiences. This may contribute to more equal access to health services and to reduced barriers to participation for those living in remote areas. Thus, both patients and local health care personnel may have a closer follow-up after initial training in the hospital. Remote monitoring centers, which are used in some countries for nightly HHD, may also be a model [18,19].

### Conclusions

In this study patients experienced a normalization of daily life, less dominated by disease when performing home dialysis. They found the treatment easy to learn, had achieved considerable self-management skills, but still needed a very strong link to the hospital for communication and follow-up. There is a need for unbiased and structured predialysis information, including access to other patients' experiences, and for organizing the treatment so that the potential for home dialysis can be better exploited. Especially for the patients with dialysis machines, telemedicine may potentially contribute to increased safety in the home setting, making it easier to choose and live with home dialysis. We suggest that telemedicine can contribute in the care for the chronically ill in general, as it may facilitate a communication-based follow-up with patients and professionals in a collaborative partnership.

## Competing interests

The authors declare that they have no competing interests.

## Authors' contributions

ER and EA contributed to the study design, performed the interviews and participated in the analysis and drafting of the manuscript. EJ contributed to the analysis of the material and to the drafting of the manuscript. MR participated in conceiving and designing the study, and participated in drafting the manuscript. All authors read and approved the final manuscript.

## Pre-publication history

The pre-publication history for this paper can be accessed here:

http://www.biomedcentral.com/1471-2369/13/13/prepub
